# Dissociation of spatial and object memory in the hippocampal formation of Japanese quail

**DOI:** 10.1016/j.isci.2022.103805

**Published:** 2022-01-22

**Authors:** Chelsey C. Damphousse, Noam Miller, Diano F. Marrone

**Affiliations:** 1 Department of Psychology, Wilfrid Laurier University, 75 University Avenue W, Waterloo, ON N2L 3C5, Canada

**Keywords:** Biological sciences, Neuroscience, Neuroanatomy

## Abstract

The mammalian temporal cortex can be functionally segregated into regions that encode spatial information and others that are predominantly responsible for object recognition. In the present study, we report comparable functional segregation in the avian brain. Using Japanese quail, we find that bilateral lesions of the hippocampus (Hp) produce robust deficits in performance in a foraging array (FA) spatial memory task, while sparing spontaneous object recognition (SOR). In contrast, lesions to the adjacent area parahippocampalis (APH) compromise both SOR and FA. These observations demonstrate a functional dissociation between Hp and APH that is comparable to the distinctions seen in mammals between the hippocampus and surrounding temporal cortex.

## Highlights

• Are spatial and object information separable in the avian hippocampal formation?
• Quail with lesions to the hippocampus are impaired in a spatial foraging task
• Lesions to area parahippocampalis also selectively impair object recognition
• Like mammals, bird hippocampus shows functional gradients in information processing

## Graphical abstract

**Figure:**
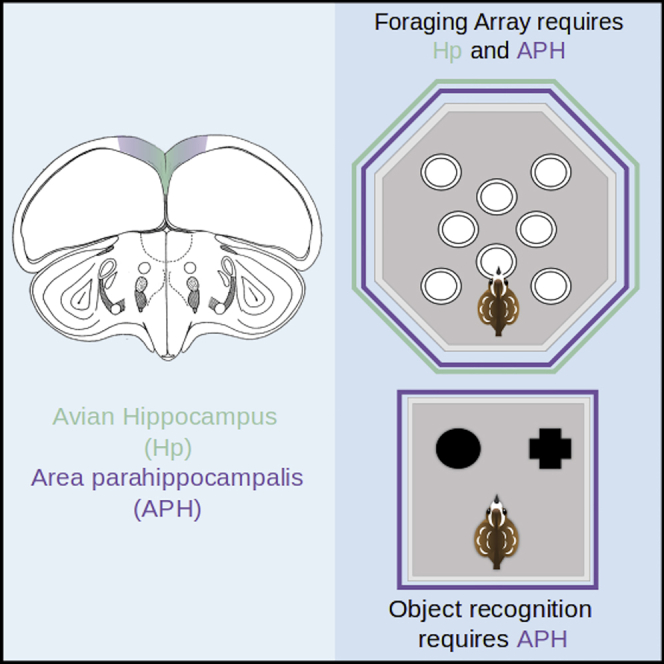

## Introduction

The hippocampus (Hp) and surrounding medial temporal lobe (MTL) structures have long been identified as critical neural circuits supporting memory, especially memory for spatial information (O’Keefe and Nadel, 1970). Recent work has shown that declarative memory can be functionally segregated both within and between structures of the MTL (Lee et al., 2017; Strange et al., 2014; Winters et al., 2004). The hippocampus and each of the surrounding cortical structures, including the entorhinal cortex (EC), make unique contributions to the computations supporting declarative memory function (see van Strien et al., 2009, for review). One important distinction is in the processing of spatial and nonspatial (e.g., object identity-based) information – with the Hp being critical to the former and often unnecessary for the latter (Eichenbaum and Lipton, 2008; Knierim et al., 2013).

The avian Hp is often proposed as the homologue to its mammalian counterpart because of similarities in development, connectivity and neurotransmitters, and because of its critical role in spatial cognition (see Székely, 1999; Colombo and Broadbent, 2000; Atoji and Wild, 2005 for review). Although many ways of dividing the avian hippocampal formation have been proposed (e.g., Erichsen et al., 1991; Montagnese et al., 1996), two methods are most commonly utilized. The first and most simplistic model describes two subdivisions, the Hp and area parahippocampalis (APH) regions (Karten and Hodos, 1967; Székely and Krebs, 1996). In the second, regions are described as the ventral (V), dorsomedial (DM), and dorsolateral (DL) subdivisions (Atoji and Wild, 2005), although these areas are often further subdivided (Atoji and Wild, 2006). Combining these two models, the Hp is largely comprised of the V and DM areas, whereas the APH corresponds to the DL. Although the inclusion of more subdivisions is more accurate, here we opt for consistency with the previous lesion studies that inform this research and refer to these areas as simply the Hp and APH. These previous studies have often ignored the boundaries between these two regions and have destroyed both Hp and APH (e.g., Good, 1987; Colombo et al., 2001; Kahn and Bingman, 2004; Broadbent and Colombo, 2000; Johnston et al., 2021). This is in part because multiple methods of dividing the avian Hp exist, and in part because of early data demonstrating that damage to either Hp or APH result in comparable spatial memory impairments (e.g., Bingman et al., 1988; Bingman and Mench, 1990). As a result, the issue of whether functional specialization might occur in different regions of the avian hippocampal formation remains largely unexplored, despite anatomical data suggesting that APH may be homologous to the EC (Redies et al., 2001; Abellán et al., 2014; Zhou et al., 2020; Bingman et al., 1994; Kröner and Güntürkün, 1999).

**Figure 1:**
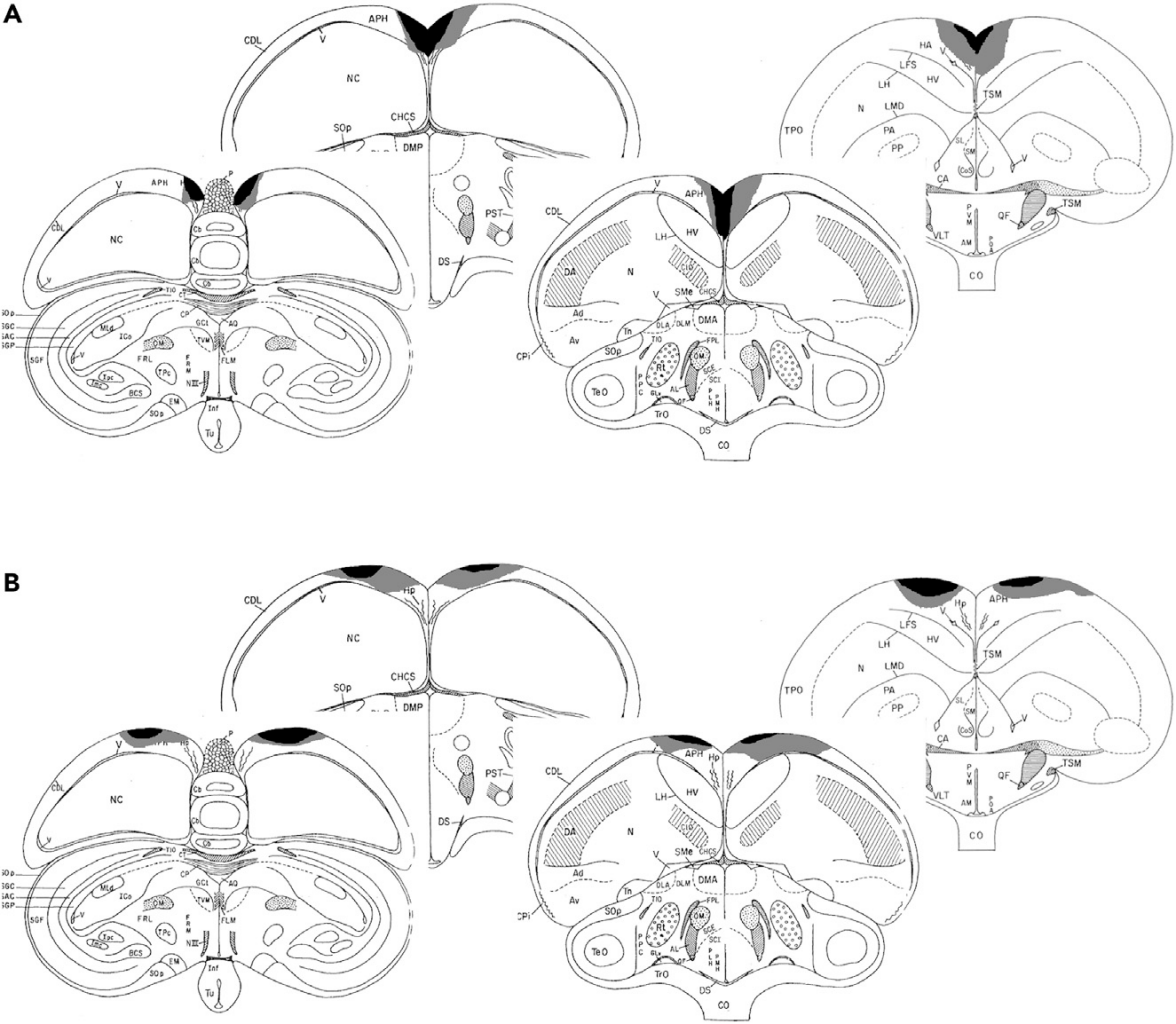
Coronal sections illustrating the extent of Hp and APH lesions Lesion reconstruction of (A) Hp-lesioned and (B) APH-lesioned quail included in the study. The black areas depict damage found in at least five of the eight lesioned quail. Gray areas show damage found in at least two of the eight lesioned quail

To address this, groups of Japanese quail (*Coturnix japonica*) underwent lesion surgery to either the APH or Hp (Figure 1) and were tested using a spatial learning task in a foraging array (FA) and a spontaneous object recognition (SOR) task, paradigms well known to require distinct structures of the mammalian memory system.

## Results

### Foraging array

**Figure 2:**
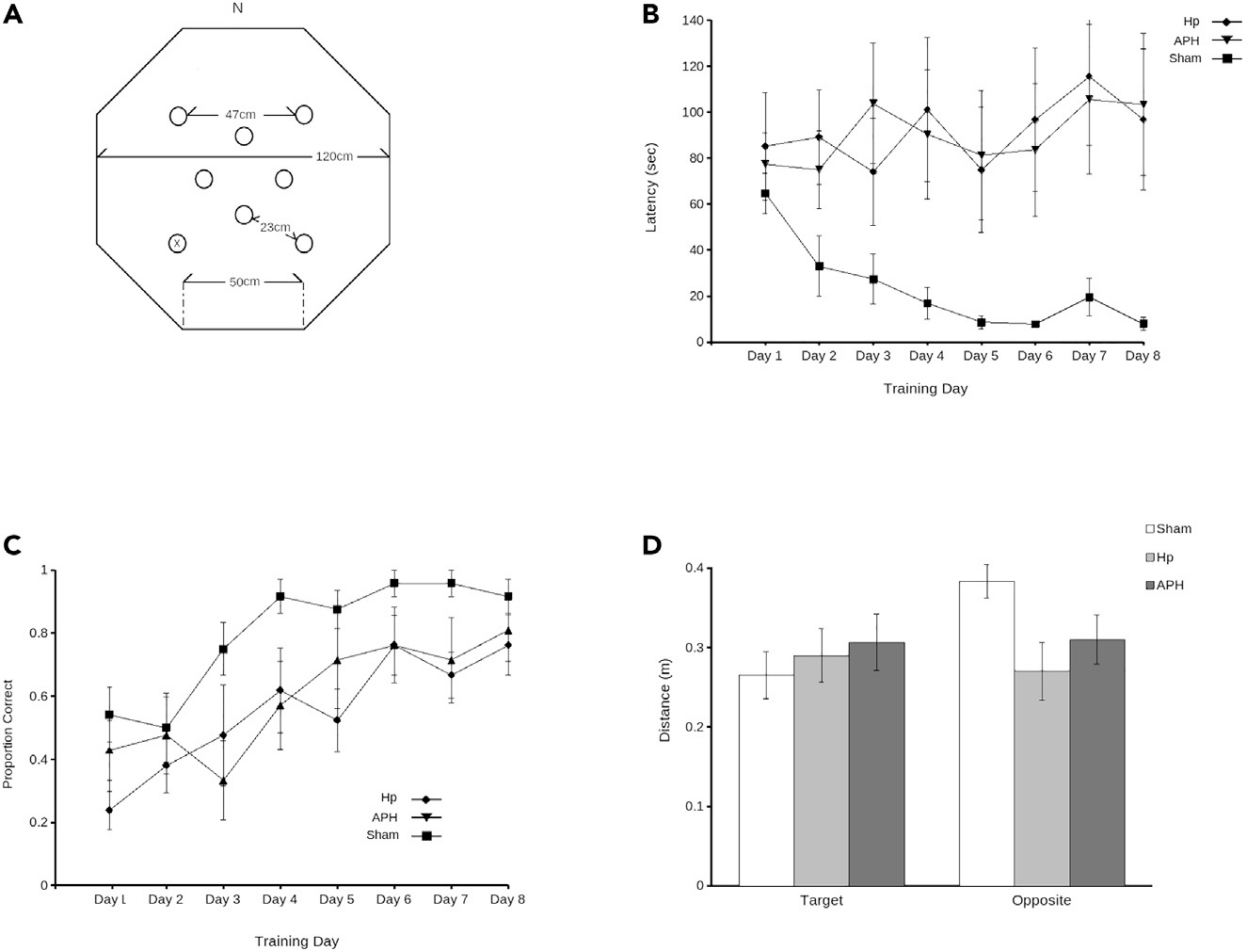
Lesions of Hp or APH impair spatial memory A schematic (A) shows the placement of reward cups including the baited cup (x) in the foraging array (FA). Calculation of latency to visit the baited cup (B) as well as first choice accuracy (C) show that intact sham quail (square) were more accurate while requiring less time to retrieve the mealworm from the baited cup relative to Hp-lesioned (diamond) and APH-lesioned (triangle) quail. Similarly, in the probe trials (D) sham quail (white) were significantly closer to the previously baited cup (target) relative to the cup on the opposite end of the arena. This was not true for Hp-lesioned (light gray) or APH-lesioned (dark gray) quail (data represent mean ± SEM).

Analysis of latency data in the FA (Figure 2B) showed no significant effect of training day (F_7,133_ = 0.55, p = 0.80) or experimental group (F_2,19_ = 2.18, p = 0.14), showing that, across the total population, there was no significant difference in latency in FA. This, however, was because two of the three groups examined showed no decrease in latency – in fact, latency increased over the 8 days of training in both lesioned groups. In contrast, the latency of intact sham quail decreased drastically over this same period, from over 60 s on Day 1 to less than 10 s on Day 8, consistent with previous observations (Lormant et al., 2018). This resulted in a significant group by training day interaction (F_14,133_ = 2.27, p < 0.01). Similarly, in *post hoc* tests, the groups did not differ (p > 0.60) on Day 1, but by Day 8 shams were significantly different (p < 0.05) from both APH and Hp lesioned birds.

However, the accuracy with which quail selected the baited cup (Figure 2C) showed a consistent increase over trials (main effect of training day: F_7,133_ = 13.87, p < 0.001) suggests that significant learning occurred in all animals. A significant difference was observed across experimental groups (F_2,19_ = 3.83, p = 0.04). Post hoc tests confirmed that this difference was the result of deficits in both lesioned groups, as both APH (p = 0.04) and Hp (p = 0.02) lesioned birds were significantly less likely to select the baited cup first relative to controls.

Consistent with these observations, analysis of the probe trial (Figure 2D) shows that sham quail spent significantly more time in the vicinity of the previously baited cup when compared to the cup on the opposite end of the arena (t_7_ = −3.52; p = 0.01). In contrast, no significant difference was observed in either Hp (t. = 0.63; p = 0.55) nor APH (t. = −0.88; p = 0.41) lesioned quail.

### Object recognition

**Figure 3:**
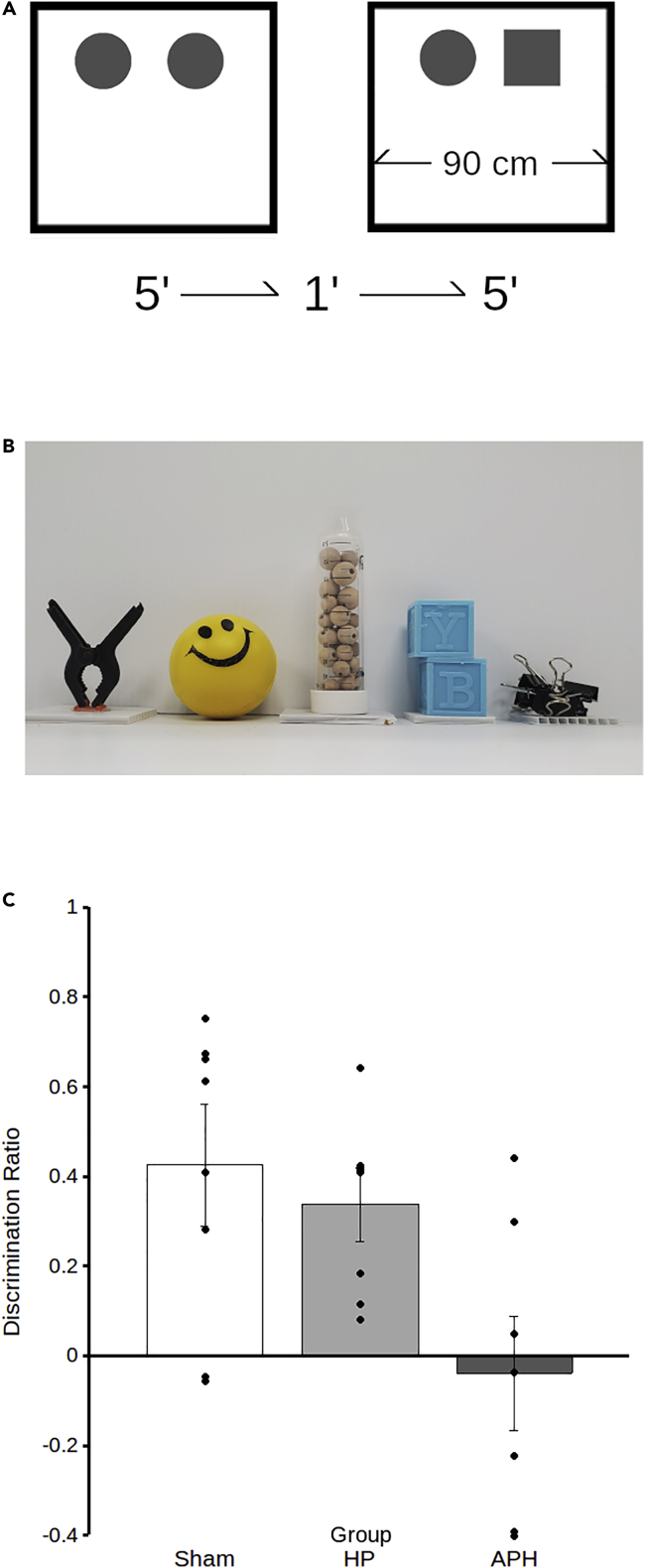
Lesions of APH but not Hp impair spontaneous object recognition (SOR) memory A schematic (A) demonstrates the placement of objects and timing of trials in SOR. Following 3 days of habituation, quail received their first sample trial (left) in an open field containing two identical copies of a novel object (circles) for 5 min. After a delay of 1 minute, quail received a choice trial (right) in which a new copy of the same object is presented alongside a novel object (square). Samples of the objects (B) used are also shown. Quantification (C) of discrimination ratios (DR) shows that intact sham quail (white) and Hp-lesioned quail (light gray) spend more time investigating the novel object, as shown by the positive mean DR. APH-lesioned quail, however, have a mean DR that is near 0, a value that reflects random chance object investigation (data represent mean ± SEM).

Analysis of SOR performance during choice trials showed a significant effect of experimental group (Figure 3; F_2,19_ = 12.95, p < 0.01) with post hoc tests showing that the performance of APH lesioned quail was significantly worse than either Hp lesioned quail (p = 0.04) or sham controls (p = 0.01). No significant differences were observed between Hp lesioned and sham quail (p = 0.59).

## Discussion

These data provide the first observation (to the authors' knowledge) of functional heterogeneity across the avian memory system that shows some consistency with the functional differentiation observed in the mammalian temporal lobe. Here we report that lesions to either APH or Hp induce robust deficits in a spatial learning task. These results are consistent with early studies in pigeons showing spatial deficits after lesions to either of these brain structures (e.g., Bingman et al., 1988; Bingman and Mench, 1990). We also report the novel observation that only lesions to APH induce a deficit in object recognition. It is worth noting here that tasks were not counterbalanced and all quail were trained in the FA task before SOR. Although counterbalancing remains the ideal, it is unlikely that practice effects from FA could alter the conclusions drawn from performance in SOR, a task with different cognitive demands that occurs in a different testing apparatus. In particular, it would be counterintuitive that any effects of practice could differentially benefit quail with Hp lesions and not those with APH lesions. Object recognition and spatial memory are tasks that rely on independent neural circuits in mammals. The current observations are consistent not only with these mammalian findings, but also with reports that damage to the avian Hp generally spare performance on visual memory tasks when careful attention is taken to minimize spatial confounds (Colombo and Broadbent, 1997; Good and Macphail, 1994; Hampton and Shettleworth, 1996). This evidence strongly suggests dissociation in the areas of the avian hippocampal formation supporting spatial cognition and object recognition.

Direct comparisons with previous literature are problematic as the nomenclature used changes frequently between papers, often with the same labels describing different regions. Given this variation in terminology, the most conservative conclusion is that the avian Hp, like its mammalian counterpart, is dispensable for object recognition memory, at least within 1 mm of the midline. In contemporary nomenclature, this certainly encompasses area V (and its subdivisions) and at least some portion of DM. Moving laterally from the midline, however, there is a point in the avian pallium (perhaps at the division between DM and DL) that also encodes nonspatial information in order to support object recognition. Given this novel observation, many questions remain to be addressed.

For instance, it remains unclear if the APH contains further functional segregation. It is perhaps surprising that APH lesions produced deficits in both spatial and nonspatial tasks, rather than producing a double dissociation between the regions responsible for object recognition and spatial cognition, as is often reported in mammals (e.g., Winters et al., 2004). There are at least two possibilities that may explain these observations. One is that the APH is less differentiated than its mammalian counterparts and contains both cells that encode spatial information and those that encode nonspatial information throughout its mediolateral extent. The presence of an undifferentiated homologue to the medial temporal cortex (equivalent to a combination of the mammalian entorhinal, perirhinal, and parahippocampal cortices) would be consistent with the lack of clear boundaries between regions of the avian hippocampal formation in general and would be consistent with previous studies that failed to find any medio-lateral gradient in spatial information content (Payne et al., 2021). A second possibility, however, is that there are gradients in the activity of APH not captured by the current protocol. The mammalian EC can be functionally separated into a lateral portion that processes nonspatial information about object identity and familiarity, whereas the medial EC specializes in spatial information (Eichenbaum and Lipton, 2008; Knierim et al., 2013). Genetic markers provide the basis for dividing the APH into a medial, intermediate, and lateral portion (Abellán et al., 2014) and the homologue of medial EC has been proposed to be the lateral division, perhaps also extending into the corticoid dorsolateral (CDL) area. In this scenario, APH lesions are likely causing spatial deficits by severing fibers of passage from the lateral APH/CDL region to the Hp, explaining why both lesion types affect performance on the spatial task (Rosinha et al., 2009; Kahn et al., 2003). It should be noted, however, that the only study to explicitly examine the behavioral effects of CDL lesions found no spatial deficits in a delayed alternation task (Gagliardo et al., 1996). These inconsistencies will require further studies using selective perturbations to disambiguate, likely in conjunction with cellular markers to more definitively differentiate regions. Despite these unanswered questions, the current data provide a new perspective on the functional heterogeneity of the avian memory system.

### Limitations of the study

The current data solely examine female quail, so potential sex differences canot be addressed. In addition, it is not known how well these data may generalize to other species of birds, particularly species better adapted to flight, as this is known to significantly influence information processing in the hippocampal formation of mammals (e.g., Finkelstein et al., 2016).

## STAR★Methods

### Key resources table

**Table:** 

REAGENT or RESOURCE	SOURCE	IDENTIFIER
**Chemicals, peptides, and recombinant proteins**		

lidocaine and epinephrine	Bimeda, Cambridge, ON	1LID010
antibacterial cleanser (Phenrex)	CDMV	
chlorhexidine gluconate solution (Baxedin)	CDMV	
Isopropyl alcohol	Sigma Aldrich, Oakville, ON	I9030-4L
Isoflurane	CDMV	
Enrofloxacin (Baytril)	CDMV	
Ketoprofen (Anafen)	CDMV	
2-methylbutane	Sigma Aldrich, Oakville, ON	270342
Nuclear fast red	Sigma Aldrich, Oakville, ON	60700

**Experimental models: Organisms/strains**		

Japanese Quail (*Coturnix japonica*)	Spring Creek Quail Farm, Saint Anns, ON	N/A

**Software and algorithms**		

ANY-maze	Stoelting	4.0
JASP	JASP team, 2021	0.16

**Other**		

SomnoSuite anesthesia system	Kent Scientific, Torrington, CT	N/A
Stereotaxic apparatus	Kopf Instruments, Tujunga, CA	Model 963
quail brain atlas	Baylé et al., 1974	
Superfrost Plus™ slides	VWR	95057-985

### Resource availability

#### Lead contact

Further information and requests for resources and reagents should be directed to and will be fulfilled by the lead contact, Diano Marrone (dmarrone@wlu.ca).

#### Materials availability

This study did not generate unique reagents.

### Experimental model and subject details

#### Animals

24 adult female Japanese quail (Spring Creek Quail Farms, Saint Anns, ON), aged approximately 3 months were used in this experiment. All birds were group housed on a 12:12 h light-dark cycle with *ad lib* access to food and water. Prior to behavioral testing, all animals were handled 15 min/day for at least 7 days. All procedures were approved by the animal care committee of Wilfrid Laurier University in accordance with the guidelines of the Canadian Council on Animal Care.

### Method details

#### Surgery

All surgeries were conducted prior to any behavioral testing. Each lesion group consisted of 8 subjects (8 APH, 8 HP, 8 Sham). Lesions (see Figure 1) were conducted in four batches of 6 (2 APH, 2 HP, 2 Sham). Each batch was tested on the FA, SOR, and sacrificed before the other batch began testing. This resulted in roughly 2 weeks between start dates for each batch.

Quail were anesthetized with isoflurane using a SomnoSuite anaesthesia machine (Kent Scientific, Torrington, CT) and placed in a stereotaxic instrument (Kopf Instruments, Tujunga, CA). Once the head was secured using ear bars and a nose cone, feathers were removed and the area was prepared using antibacterial cleanser (Phenrex®), 70% isopropyl alcohol, and chlorhexidine gluconate solution (Baxedin®). Following subcutaneous injection of lidocaine and epinephrine (Bimeda, Cambridge, ON) along the midline of the skull, a midline incision was made, the scalp was retracted, and a craniotomy was made over the lesion site (1 craniotomy for Hp lesion, 2 for APH). The Hp and APH were removed by aspiration according to coordinates determined using a published quail brain atlas (Baylé et al., 1974). Coordinates for lesions were determined relative to where the parieto-occipital suture intersects with the midline. For Hp lesions, aspirations were 5 mm anterior to bregma, 3 mm posterior, 1.5 mm on either side of midline, and 3 mm deep. Aspirations for APH lesions were 5 mm anterior to bregma, 3 mm posterior, 1.5 mm – 3.5 mm lateral to bregma, 2 mm deep.

Craniotomies were packed using a hemostatic sponge, sealed with bone wax and the skin was sutured. After recovering on a heating pad and regaining mobility, quail were placed into individual cages to recover for 1 week while undergoing antibiotic and analgesic treatment.

#### Foraging array

The FA (see Figure 2A) followed testing methods previously described by Lormant et al. (2018). Briefly, an octagonal arena (each wall 50 cm in length, 45 cm in height) was constructed using white corrugated plastic sheeting. The flooring was also corrugated plastic sheeting. Eight unique visual cues constructed of black poster board cut into 8 unique geometric shapes were used in the arena. Four cues were placed as local cues on walls within the maze and the other four were used as distal cues attached to the walls of the room near the ceiling so that they were visible to subjects within the arena. Eight food cups were placed in the arena in the configuration depicted in Figure 2A. Food cups were constructed using a 2 oz plastic cup, with a 1.5 oz cup with a perforated bottom nested within it. The outer cup contained inaccessible mealworms in order to control for scent cues.

The FA consisted of three phases: habituation, training, and probe. 1 hr prior to beginning all phases of the experiment, food was removed and subjects were transported to the testing room in a rack containing all subjects in individual cages. The rack was surrounded by a curtain and subjects were left undisturbed. There were 5 days of habituation in total. The first 2 days were habituation to transport. Subjects were transported into the testing room from their homeroom and left undisturbed for 1 hr. Over the next 3 days, subjects were habituated to the arena. During arena habituation, subjects were placed into the centre of the arena with all cups baited with one mealworm. Sessions were recorded using an overhead webcam and number of mealworms eaten was scored. Subjects were removed once all mealworms had been consumed or after 600 sec had elapsed.

Subjects received 3 training trials per day (1 hr ITI) over the course of 8 days. During training, only one cup was baited (SW) and this remained consistent throughout all of the training trials. Subjects were placed into the maze at 1 of 3 locations (N, S, E) chosen at random for each trial. Trails were 300 sec in duration or until the subject had retrieved worms from the baited cup. Latency to reach the cup was recorded using ANY-maze tracking software. After 8 days of training trials, subjects underwent a probe trial in which none of the cups were baited and subjects entered the maze from a novel direction (W).

#### Spontaneous object recognition

The SOR protocol used here was adapted from a previous publication testing Japanese quail (Damphousse et al., 2021). Briefly, in a second testing room, birds underwent two SOR tests over two consecutive days within a square arena with walls 90 cm wide and 60 cm tall, constructed from painted plywood (see Figure 3A). Flooring was corrugated plastic sheeting. A spatial cue was placed onto one wall of the arena. Behaviour was monitored using an overhead webcam and tracking was done in real time using ANY-maze. All subjects encountered the same sets of objects, with object sets differing between test days. Identical to the spatial learning task, food was removed, and subjects were left undisturbed in a covered rack for 1 hr prior to beginning the experiment. Subjects were habituated to the empty testing arena over 3 consecutive days for 300 sec per day. On the first day of testing, subjects underwent a sample phase immediately followed by test. During the sample trial, two identical junk objects were placed into the arena in the centre of the two quadrants furthest from the entry point. Subjects explored the objects and arena for 300 s. The subject was removed and over the course of a 1 min ITI, the choice trial was prepared by placing an object identical to those used during sample (familiar) and a novel object within the arena. The arena was also wiped down with 70% Ethanol to eliminate scent trails and the subject was placed into the arena to explore for 300 s. Exploration was defined as the bird spending time within 30 cm of an object while not preening or pecking at the surrounding walls. The entire body of the subject was to be within the defined 30 cm radius and orientation of the subject toward the object was not required as the field of vision for prey birds, such as quail, is large and an object may be viewed from many positions relative to the head (Martin and Young, 1983; for review see Martin and Osorio, 2008). This criterion successfully demonstrates novelty preference in multiple avian species, including Japanese quail (Damphousse et al., 2021). While 30 cm is a generous distance, quail have much better visual acuity (4.73 ± 0.35 c/d; Lee and Djamgoz, 1997) than albino strains of rat (0.5 c/d; Prusky et al., 2002) used within protocols from which the SOR task was originally adapted (for review see Blaser and Heyser, 2015). The time spent exploring the novel (N) and familiar (F) objects for all birds was converted into a discrimination ratio (DR) as follows: DR = (N - F) / (N + F). On the second day of testing, an identical procedure was followed using a second, visually distinct set of objects.

#### Histology

Following SOR, 25 days post-surgery for a given batch, subjects were transported to a procedure room, anesthetized using isoflurane, decapitated, and brains were extracted and flash frozen in 2-methylbutane (Sigma Aldrich, Oakville, ON). Coronal sections were cut at a thickness of 30 μm using a CM3050 cryostat (Leica), thaw-mounted onto Superfrost Plus™ slides (Thermo Scientific, Waltham, MA), dried, and stored at -80°C. Every 6th section was then stained using Nuclear fast red-aluminum sulfate to observe placement and extent of the lesions under a light microscope.

### Quantification and statistical analysis

In the FA task, the mean latency to reach the target cup as well as the number of trials in which the baited cup was calculated for each dat of training. These data were analysed using a repeated measures analysis of variance (ANOVA). Lesion location was the between subject factor and training day was the within subject factor. The probe trials were analysed by comparing the mean proximity of each quail to the previously baited cup relative to the cup on the opposite side of the maze using a paired t-test within each group. In SOR, the mean DR for each quail across both object sets was compared using a one-way ANOVA for lesion location. All statistical analyses were conducted using JASP (JASP team, 2021) using Tukey’s HSD in all post hoc tests.

## Data Availability

• All data reported in this paper will be shared by the lead contact upon request. • This paper does not report original code. • Any additional information required to reanalyse the data reported in this paper is available from the lead contact upon request.
